# Associated risk factors for psychological distress in patients with gastric epithelial neoplasm undergoing endoscopic submucosal dissection

**DOI:** 10.1097/MD.0000000000013912

**Published:** 2018-12-28

**Authors:** San Lee, Seung-Taek Oh, Hyeok Lee, Jae Seung Lee, Haeyong Pak, Won-Jung Choi, Han Ho Jeon

**Affiliations:** a Department of Psychiatry; b Department of Internal Medicine; c Institute of Health Insurance and Clinical Research, National Health Insurance Service Ilsan Hospital; d Yonsei Hana Psychiatry Clinic and Institute of Mental Health, Goyang; e Department of Psychiatry and Institute of Behavioral Science in Medicine, Yonsei University College of Medicine, Seoul, Korea.

**Keywords:** anxiety, depression, distress, endoscopic submucosal dissection, gastric epithelial neoplasm

## Abstract

This study aimed to evaluate the psychological distress and associated risk factors for distress among patients with gastric epithelial neoplasm undergoing endoscopic submucosal dissection (ESD).

A total of 91 patients treated with ESD for gastric epithelial neoplasm between May 2015 and June 2016 were prospectively enrolled. Sociodemographic factors, psychological distress, anxiety, depression, stress, and associated risk factors for psychological distress were evaluated the day before ESD.

Twenty-six (28.6%) patients were identified as patients with psychological distress. The psychological distress group had a higher female ratio and more depression and anxiety symptoms than the non-distress group. Distress was also related to stress level. A multivariate analysis showed that unmarried status (odds ratio [OR], 4.94; 95% confidence interval [CI], 1.13–21.56, *P* = .034), anxiety (OR, 1.24; 95% CI, 1.12–1.39, *P* <.001), and stress (OR, 1.06; 95% CI, 1.01–1.12, *P* = .011) were associated with psychological distress.

An unmarried status and a high level of anxiety and stress were associated with more psychological distress in patients undergoing gastric ESD. It could be helpful to screen and proactively monitor patients with such conditions before performing gastric ESD.

## 1 Introduction

The overall cancer survival rate has improved continuously due to constant advances in the preventive, diagnostic, and therapeutic modalities of cancer treatment. Nevertheless, many cancer patients still regard a cancer diagnosis itself as a stigma of incurable disease, and many of them suffer from psychological distress throughout various stages (from cancer diagnosis to end-stage treatment).^[1,2]^

The National Comprehensive Cancer Network (NCCN) has defined distress as a multi-factorial, unpleasant experience of an emotional, psychological, social, or spiritual nature that interferes with the ability to cope with cancer, its physical symptoms, and its treatment.^[3]^ Distress can lower therapeutic compliance^[4]^ and negatively affect the survival rates of cancer patients.^[5]^ Furthermore, distress can reduce patient quality of life.^[6]^ Because of these features, many experts in cancer treatment insist that distress should be regarded as a sixth vital sign.^[7]^ Therefore, it is important to screen for psychological distress and to intervene properly at every step of cancer evaluation and treatment.

In patients with gastric epithelial neoplasm (including gastric dysplasia or early gastric cancer), endoscopic submucosal dissection (ESD) is considered standard treatment. Even if a lesion is large, ESD makes it possible to do en bloc resection and to evaluate the histology of totally excised specimens. Improvement of endoscopic devices has occurred, but ESD is still a technically difficult procedure that requires more time than endoscopic mucosal resection (EMR). Complications such as pain, bleeding, and perforation may occur and surgical re-treatment is sometimes necessary based on the pathology of the excised specimen. Therefore, a patient who is planning to undergo gastric ESD may encounter psychological distress, and it is necessary to intervene appropriately in such a case.

To the best of our knowledge, there has been no study to evaluate distress, anxiety, and depression in patients with gastric epithelial neoplasm undergoing gastric ESD until now. The present study aimed to prospectively evaluate the prevalence of psychological distress and its associated risk factors in patients scheduled for ESD to remove gastric epithelial neoplasm.

## 2 Patients and methods

### 2.1 Patients

Patients with gastric epithelial neoplasm (including gastric dysplasia or early gastric cancer) were prospectively enrolled at the National Health Insurance Service Ilsan Hospital in Korea from May 2015 to June 2016. All patients were of Eastern Cooperative Oncology Group (ECOG) performance status 0 or 1. They were classified using the American Society of Anesthesiologists (ASA) physical status classification system. Each patient had gastric dysplasia or early gastric cancer that had been diagnosed by previous biopsy. Patients who underwent subtotal gastrectomy or gastrostomy, those with more than 1 lesion, and those who needed a second ESD were excluded from this study. Patients with neurological disease, cognitive impairment, or difficulty reading and understanding written informed consent were also excluded. The Institutional Review Board of National Health Insurance Service Ilsan Hospital reviewed and approved our study protocol (NHIMC 2015-03-013-001), and we obtained written informed consent from all patients. The ClinicalTrial.gov registration number is KCT0001792. All ESD procedures were performed by a single endoscopist (Jeon). Therefore, the explanation of ESD (current diagnosis, ESD procedure, success rate, complications as like bleeding and perforation, pain and prognosis) for all patients was done by the same clinician.

### 2.2 Data collection

Patients were admitted the day before their gastric ESD procedure. On the day of admission, patients were given oral and written information about the study aims and procedure. If patients were willing to participate, they received an informed consent form. After signing the informed consent, baseline characteristics (including sociodemographic factors) were evaluated for each patient. Because the Montgomery-Asberg Depression Rating Scale (MADRS) and the Hamilton Anxiety Rating Scale (HAM-A) are clinician-rating scales, the scales were administered by trained psychiatrists at the hospital. Other self-report scales for distress, anxiety, and stress were also administered on the day before gastric ESD.

### 2.3 Measures

Three self-report scales and 2 clinician-rating scales were used in the study. The self-report scales (including the distress thermometer (DT) for distress, the Global Assessment of Recent Stress (GARS), and the Perceived Stress Scale (PSS) for stress were administered before gastric ESD. The MADRS and the HAM-A were assessed by trained psychiatrists on the same day.

The DT was developed by NCCN as a self-report scale for measuring the distress of cancer patients.^[8,9]^ The scale asks about the severity of psychologically unpleasant experiences during the past week. The distress level is quantified using a visual analog scale ranging from 0 to 10. A distress score of 4 is considered the cut-off score with optimal sensitivity and specificity in a general cancer population.^[10]^ We also defined patients with distress score of 4 or more as distress group in this study. The GARS scale evaluates correlations between the present physiologic state and stress.^[11]^ It consists of 8 items, and a higher score reflects more stressful conditions. The PSS shows subjective perceptions of stress.^[12]^ It focuses on the usual situational context rather than on specific event experiences.

The MADRS includes 10 items related to depression.^[13]^ Each item is measured by a clinician using a Likert scale ranging from 0 to 6. The scale covers cognitive, affective, and biological dimensions of depression. The HAM-A was developed to assess 14 items.^[14]^ It is performed by a clinician using a semi-structured interview, and the output consists of 2 factors: a factor of general psychiatric anxiety symptoms, and a factor of cognitive and somatic symptoms. Matza et al. reported scores in a study of patients with generalized anxiety disorder as mild (8–14), moderate (15–23), and severe (24 or more).^[15]^

### 2.4 Statistical analysis

A sample size of 72 achieves 81% power to detect a ratio (P1/P0) of 0.6 using a 2-sided binomial test. The target significance level is 0.05. The actual significance level achieved by this test is 0.0402. These results assume that the population proportion under the null hypothesis is 0.4.

We calculated percentages and mean values for sociodemographic, clinical, and psychiatric variables. The independent *t*-test and chi-square test were used to compare differences in sociodemographic, clinical, and psychiatric factors. Logistic regression analysis was performed to estimate the odds ratio (OR) and 95% confidence intervals (CIs) for factors that were independently associated with distress. *P* values below .05 were considered statistically significant and the data analysis was performed with SPSS version 23 (IBM Corp., Armonk, NY).

## 3 Results

### 3.1 Patient characteristics

As shown in Table 1, 56 (61.5%) of the patients were male. Most patients (81.3%) reported that they were married. An analysis of ASA physical status showed that 71 (78.0%) were classified as group 1 or 2. Ten patients (11.0%) had psychiatric comorbidities. The gastric epithelial neoplasm lesion was antrum in 57 cases (62.6%), and the size of most lesions (76.9%) was 10 to 20 mm. Eighty-one patients (89.0%) received a diagnosis of dysplasia before ESD, and ten patients (11.0%) were diagnosed with early gastric cancer before ESD. The mean scores for depression (MADRS), anxiety (HAM-A), and stress perception (GARS) were 8.53, 8.07, and 16.97, respectively. Detailed data are shown in Table 1.

**Table 1:**
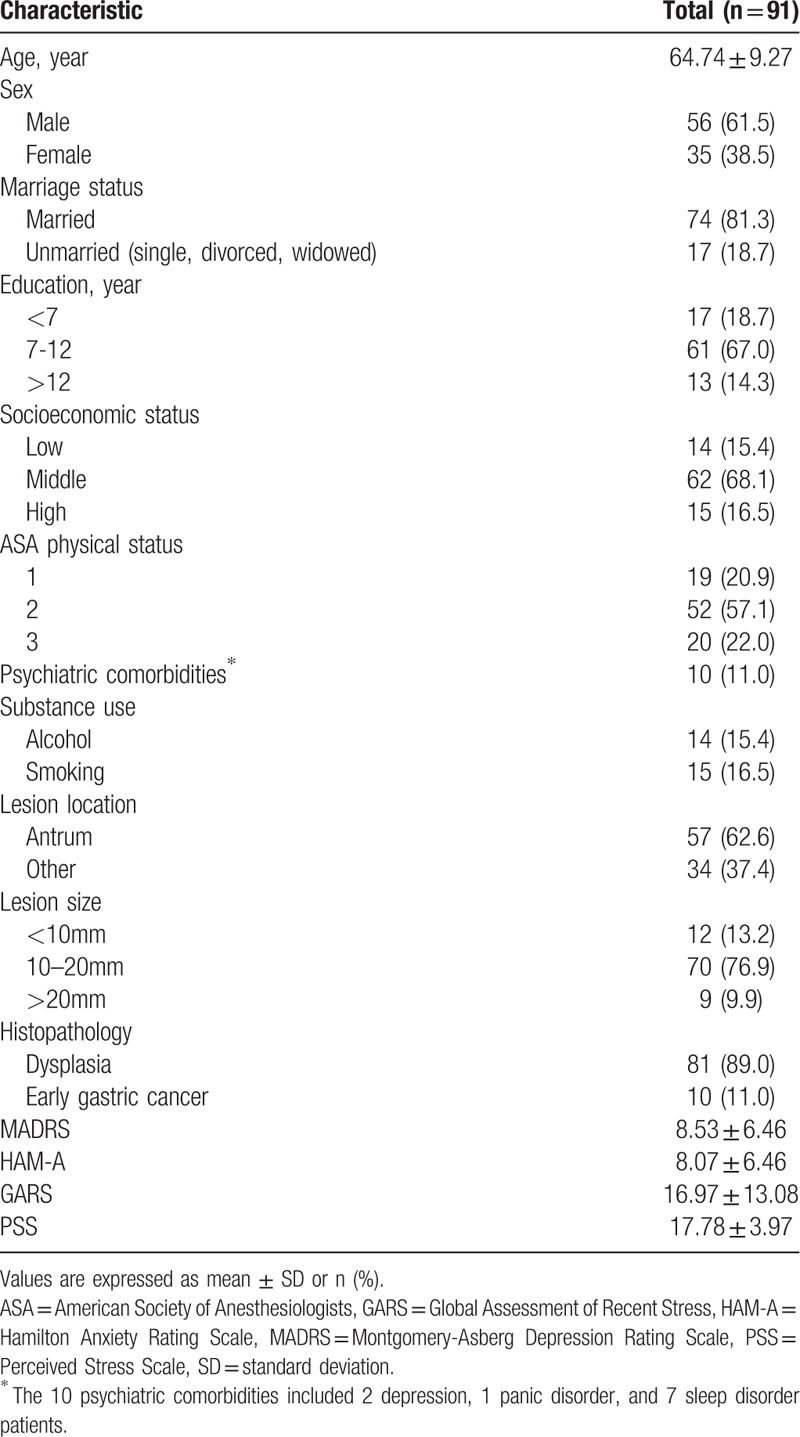
Patient baseline characteristics.

### 3.2 Comparison between distress and non-distress groups

Table 2 shows a comparison of sociodemographic and clinical factors for patients with and without psychological distress, using a preset distress cut-off score of 4. Among 91 patients, 26 (28.6%) were identified as patients with psychological distress. The distress and non-distress groups showed no difference in age, education level, or socioeconomic status. The groups also did not differ in ASA physical status, alcohol and smoking history, lesion location, lesion size, or histopathologic findings before ESD. The distress group showed higher female and higher unmarried status ratios than the non-distress group (*P* = .004, *P* = .014, respectively). Psychiatric comorbidity was more common in the distress group (*P* = .020). Depressive and anxiety symptom scores measured by MADRS and HAM-A differed significantly between the groups, with higher symptom scores in the distress group (*P* <.001, *P* <.001, respectively). The stress scales of GARS and PSS also revealed that the distress group had more general stress than the non-distress group (*P* <.001, *P* =.001, respectively).

**Table 2:**
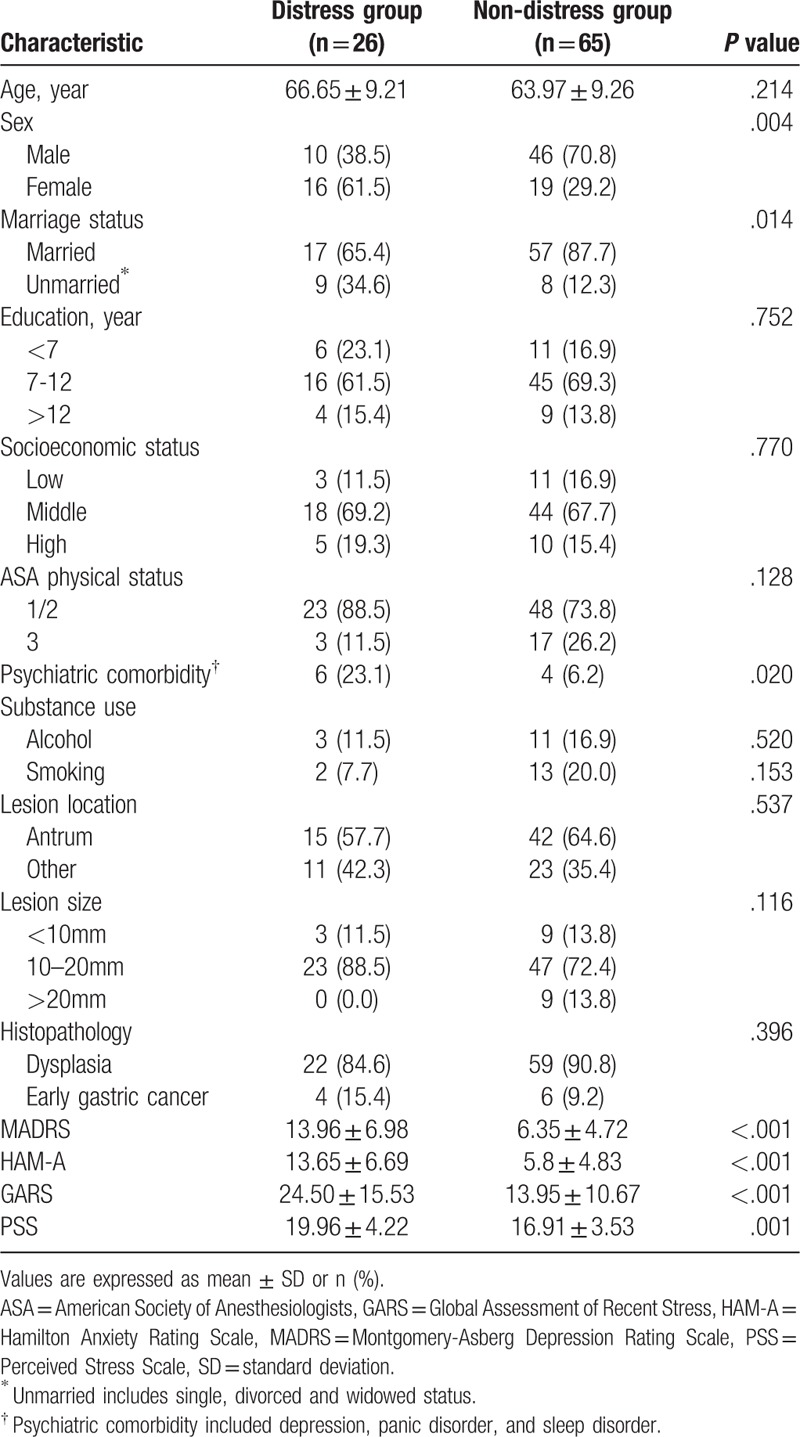
Comparison between distress and non-distress groups.

### 3.3 The risk factors of psychological distress

A logistic regression analysis was performed to identify the risk factors of psychological distress (Table 3). Univariate logistic regression analysis revealed variables showing a significant correlation with distress included female sex, unmarried status, psychiatric comorbidity, depression (MADRS), anxiety (HAM-A), and stress (GARS). A multivariate logistic regression analysis of distress showed that unmarried status (OR, 4.94; 95% CI, 1.13–21.56, *P* = .034), anxiety (HAM-A) (OR, 1.24; 95% CI, 1.12–1.39, *P* < .001), and stress (GARS) (OR, 1.06; 95% CI, 1.01–1.12, *P* = .011) were risk factors related to psychological distress.

**Table 3:**
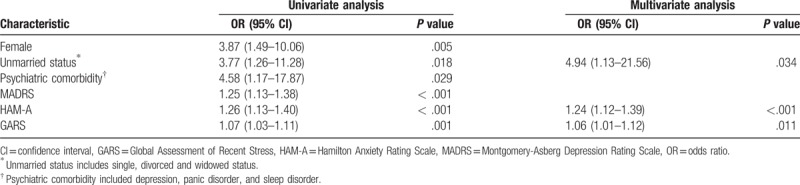
Univariate and multivariate logistic regression analyses of distress.

## 4 Discussion

In this study, the prevalence of psychological distress in patients with gastric epithelial neoplasm undergoing ESD was 28.6%. The prevalence was relatively lower than that reported in previous studies of patients with gastric cancer in Korea.^[16,17]^ In the previous studies, enrolled patients were diagnosed with gastric cancer of various stages before undergoing distress evaluations. In contrast, we enrolled only patients with dysplasia or early gastric cancer, diagnoses which call for ESD. Therefore, the previous studies included more severe cases of gastric cancer compared with our study. In one of the previous studies, distress was not only evaluated using the Modified DT but also with the Hospital Anxiety and Depression Scale (HADS) and the Center for Epidemiologic Studies-Depression Scale (CES-D) (which are used globally to check levels of anxiety or depression). It is possible that the different patient inclusion criteria and distress evaluation scales account for the difference in the prevalence of distress.

When comparing the distress group and the non-distress group, it was evident that females felt more distress than males. Thus, the gender difference for distress seen in previous studies across various cancer types was identified again in this study.^[10,16,18–20]^ More emotional and less rational coping styles in women might influence the degree of the distress experience.^[21,22]^ Unmarried status was also found to be related to distress. This might imply the importance of the basic social support of marriage when a patient faces a cancer diagnosis and treatment. However, socioeconomic status (another indication of social support) was not related to distress. There are several possible reasons for these differences. ESD is available in almost every region of Korea. Furthermore, ESD is covered by medical insurance. It might be that the economic burden of gastric ESD was light. Depression (measured by MADRS) and anxiety (measured by HAM-A) were found to be related to distress. Several studies have used HADS as a criterion measure to validate the DT among cancer patients.^[23–25]^ This may reflect the association between depression, anxiety, and distress, and our results also replicated this association. The scales for evaluating general distress perceptions (GARS and PSS) were also found to have clinical relevance. In other words, patients with distress feel much more psychological discomfort. This is true not only for cancer-specific events but for stressful events in daily life.

Our study showed related factors affecting patient experiences of distress. Unmarried status, a high level of anxiety, and a general stress perception were identified as factors contributing to distress. Distress, by definition, is the personal and subjective level of discomfort associated with cancer diagnosis and treatment. The finding that unmarried status (and therefore lack of a major social support system) can contribute to distress suggests that clinicians should pay increased attention to unmarried patients, and this result is consistent with previous studies on various types of cancer.^[26]^ Anxiety measured by the HAM-A scale was also a factor affecting distress. One prior study has shown that anxiety is a significant psychological state that contributes to a feeling of distress in breast cancer patients, and our findings are consistent with that result.^[27]^ HAM-A, the clinician-rating anxiety scale, covers both psychological aspects of anxiety and somatic symptoms of the cardiovascular, respiratory, genitourinary, and autonomic nervous systems. It is possible that this clinician-rating anxiety scale is, therefore, more suitable to evaluate the anxiety of patients who suffer from concurrent somatic symptoms. Using the scale to screen patients before gastric ESD could help to establish which patients are prone to feel anxiety and distress, and this might be useful to develop structured interventions for them. Stress identified by GARS but not PSS was also shown to be a factor influencing distress. The PSS scale includes an evaluation of coping skills for stress, but the GARS scale highlights a general stress perception in and of itself. This result may reflect shared aspects between perception of stress and distress. There is no optimal cutoff value on the GARS scale for stress. Therefore, if a patient self-reports considerable stress and the GARS analysis shows moderate to severe stress conditions, it is best to consider further evaluations or interventions for that patient.

Early detection of the psychological distress will be possible in the screening process, and it can provide the potential to intervene properly not only in psychological distress but also in other psychiatric comorbidities which the patients might have. More detailed explanation and focused examination of somatic symptom can be applied during the treatment to lower the distress because high anxiety level measured by HAM-A scale reflect much more somatic symptoms of anxiety. In addition, the proper consultation using the screening process could help the patient with proper emotional support during the treatment. To summarize, screening before gastric ESD and considering further psychiatric evaluation for patients with the risk factors discussed above could improve personal satisfaction with the procedure and lower patient distress.

There are several limitations in our study. First, this study is the cross-sectional nature of the design. It is necessary to undertake further, extended prospective longitudinal studies to evaluate the distress of patients with early gastric cancer and precancerous lesions. Second, though the explanation of ESD was done using same explanatory materials according to the biopsy-proven diagnosis before ESD, 1 endoscopist was in charge to explain and perform the ESD procedure in our study. Because how to explain the disease and planned ESD procedure to patients is one of factors that may affect the patients to feel psychological distress.

In conclusion, this study revealed that an unmarried status and high levels of anxiety and stress were associated with more psychological distress in patients undergoing gastric ESD. It could be helpful to screen and further evaluate patients experiencing such conditions before performing gastric ESD, which may improve subjective therapeutic satisfaction and lower distress during gastric ESD.

## Author contributions

**Formal analysis:** Haeyong Pak.

**Supervision:** Seung-Taek Oh, Hyeok Lee, Jae Seung Lee.

**Writing – original draft:** San Lee.

**Writing – review & editing:** Won-Jung Choi, Han Ho Jeon.

Han Ho Jeon orcid: 0000-0002-3393-3304.
